# Interaction among activated lymphocytes and mesenchymal cells through podoplanin is critical for a high IL-17 secretion

**DOI:** 10.1186/s13075-016-1046-6

**Published:** 2016-06-23

**Authors:** Mélissa Noack, Ndiémé Ndongo-Thiam, Pierre Miossec

**Affiliations:** Immunogenomics and Inflammation Research Unit, EA 4130, Edouard Herriot Hospital, Hospices Civils de Lyon and University Claude Bernard Lyon 1, Place d’Arsonval, Lyon, 69003 France

**Keywords:** IL-17, Synoviocytes, Rheumatoid arthritis, Cell interactions, Podoplanin

## Abstract

**Background:**

During chronic inflammation, immune cells, notably Th17 cells, infiltrate the inflammatory site and interact with local mesenchymal cells. Applied to rheumatoid arthritis (RA), the aim is to study the interactions between synoviocytes and peripheral blood mononuclear cells (PBMC) with a focus on the Th17 pathway and to identify a mechanism which leads to high IL-17 secretion with an interest on podoplanin.

**Methods:**

PBMC from healthy donors and RA patients were co-cultured with RA synoviocytes during 48 h, in the presence or not of phytohemagglutinin. An antibody against podoplanin was used in co-culture. Cytokine production (IL-6, IL-1β and IL-17) was measured by ELISA and cell staining (CD3, CD4, IL-17 and podoplanin) by flow cytometry.

**Results:**

In control conditions, IL-6 and IL-1β production was increased in PBMC-synoviocyte co-culture compared to PBMC alone (*p* = 0.02). No additional effect was observed with PBMC activation. Flow cytometry analysis showed no difference in the percentage of Th17 cells in activated PBMC alone or with synoviocytes (*p* = 0.4), indicating that Th17 differentiation requires only T cell activation. Conversely, IL-17 production was highly increased in co-cultures with activated PBMC vs. activated PBMC alone (*p* = 0.002). Transwell experiments confirm that cell-cell contact was critical for IL-17 secretion. The incubation of either PBMC or synoviocytes with an anti-podoplanin antibody decreased IL-17 secretion by 60 % (*p* = 0.008).

**Conclusions:**

Interactions between resting PBMC and synoviocytes are sufficient to induce IL-6 and IL-1β production. Both PBMC activation and cell interactions are needed to induce a high IL-17 secretion. Podoplanin contributes at the level of both lymphocytes and synoviocytes.

## Background

Interleukin-17 (IL-17) is a pro-inflammatory cytokine mainly produced by Th17 cells and involved in several chronic inflammatory diseases, such as psoriasis or rheumatoid arthritis (RA) [1, 2]. Commonly, an association is made between Th17 cells and the secretion of IL-17, using intracellular staining of IL-17 as an equivalent of actual demonstration of protein secretion. As shown in one of our previous studies, IL-17-positive cells acquire a plasma cell-like morphology under in vitro activation, which is correlated with enhanced concentrations of secreted IL-17. Moreover, in sections of inflamed tissue, IL-17+ cells have this plasma cell-like morphology [3]. The connection between IL-17 intracellular staining and IL-17 secretion thus remained to be clarified.

During chronic inflammatory diseases, the recruitment of activated immune cells, including Th17 cells, to the site of disease, leads to interactions with local mesenchymal cells and this promotes cell proliferation and the chronicity of inflammation [4–6]. These interactions between bone marrow-derived or synovium-derived mesenchymal cells and activated peripheral blood mononuclear cells (PBMC) promote Th17 cell expansion [7].

Regarding the crucial role of cell–cell interactions in pro-inflammatory cytokine production, a mechanism remained to be identified. Among many options, podoplanin (pdpn) was considered as a possible candidate, as a molecule involved in cell–cell interactions. Indeed, pdpn modulates IL-8 secretion during interactions between platelets and synoviocytes through its receptor CLEC-2 on platelets [8]. Moreover, the pdpn pathway is involved in different animal models of inflammatory diseases [9, 10]; notably in a mouse model of RA, pdpn is upregulated in Th17 cells compared to other Th cell subsets [9]. Pdpn expression is also upregulated in RA synovium and it might increase the migratory potential of activated synoviocytes in RA [11]. These results lead us to consider pdpn as a potential new mechanism involved in the Th17 pathway during chronic inflammation.

The aim of this study was to clarify the difference between intracellular expression from IL-17 secretion and to identify a mechanism which leads to high IL-17 secretion during interactions between PBMC and synoviocytes, with a special interest in podoplanin.

## Materials and methods

### Samples

RA synoviocytes were obtained from synovial tissue of RA patients undergoing joint surgery and who fulfilled the American College of Rheumatology criteria for RA [12]. Synovial tissue was minced into small pieces and then adhered in 6-well plates in Dulbecco’s modified Eagle’s medium (DMEM; Eurobio, Courtaboeuf, France) supplemented with 10 % fetal bovine serum (FBS; Life Technologies, Carlsbad, CA, USA), 2 mM L-glutamine and 100 U/ml penicillin/streptomycin. Cells were maintained at 37 °C in a humidified 5 % carbon dioxide incubator and used between passages 4 to 9. They are fibroblast-like synoviocytes which express pdpn and secrete IL-6 after stimulation by tumor necrosis factor (TNF) [11, 13]. PBMC from healthy donors or RA patients were isolated by Ficoll-Hypaque (Eurobio, Courtaboeuf, France) density-gradient centrifugation. Adipose-derived stem cells (ASC) and human umbilical vein endothelial cells (HUVEC) were used as control cells.

### T cell cloning procedure

To obtain CD4+ T cell clones, CD4+ T cells were isolated from peripheral blood mononuclear cells (PBMC) of healthy donors by immunomagnetic cell separation (Miltenyi Biotech, Bergisch Gladbach, Germany) and further divided into the CD161 + CCR6+ and CD161-CCR6- fractions by flow cytometry cell sorting (FACSAria, BD Bioscience, Franklin Lakes, NJ, USA). Both cell fractions obtained were seeded under limiting-dilution conditions (0.5 cell/well) in round-bottom microwell plates, containing 10^5^ irradiated (60 Gy [9000 rad]) allogeneic peripheral blood mononuclear cells as feeder cells, 1 % phytohemagglutinin (PHA; vol/vol), and recombinant human IL-2 (50 U/mL). Blasts were expanded in the presence of feeder cells (10^5^ cells/well) plus IL-2 (50 U/mL). T cell clones were obtained and evaluated for their cytokine production activity (interferon [IFN]-γ, and IL-17) by flow cytometry after stimulation with PMA plus ionomycin, as previously described [14, 15]. T cell clones producing IL-17 alone were classified as Th17. Production of cytokines by each clone was arbitrarily considered as significant when the proportion of producing T cell blasts was >20 %. Th17 clones obtained from the CD161 + CCR6+ fraction were selected and further expanded in the presence of feeder cells plus IL-2 until they reached the number of approximately 10^6^ blasts/clone, then they were transferred in 24-well plates and maintained in culture in the presence of IL-2 (50 U/mL).

### Co-culture assays

Co-culture was initiated by seeding RA synoviocytes, ASC or HUVEC overnight in 96-well plates at a density of 2 × 10^4^ cells/well in RPMI 1640 medium (Eurobio, Courtaboeuf, France) supplemented with 10 % human AB serum, 2 mM L-glutamine and 100 U/ml penicillin/streptomycin (complete RPMI). The next day, PBMC (1 × 10^5^ cells/well) or Th17 clones (2 × 10^4^ cells/well) were seeded in complete RPMI corresponding to 5:1 ratio or 1:1 ratio, respectively, in the presence or absence of phytohemagglutinin (PHA, 5 μg/ml). After 48 h, supernatants and cells were collected for analysis.

### Transwell assay

RA synoviocytes were seeded in 24-well plates at a density of 1 × 10^5^ cells/well in complete RPMI. After overnight incubation, PBMC were added directly on top of synoviocytes or in a cell culture insert (0.4 μm pore size) at a concentration of 5 × 10^5^ cells/well, in the presence or absence of PHA (5 μg/ml). After 48 h, supernatants and PBMC were recovered for analysis.

### Cell fixation

Synoviocytes or PBMC were fixed for 1 h, at 4 °C, in phosphate-buffered saline (PBS) 0.01 % paraformaldehyde before co-culture.

### Enzyme-linked immunosorbent assays (ELISA)

IL-17A, IL-6, and IL-1β productions were evaluated from culture supernatants with commercially available DuoSet ELISA kits, according to the manufacturer’s instructions (R&D Systems, Minneapolis, MN, USA).

### Flow cytometry

Allophycocyanin (APC), phycoerythrin (PE), PE-cyanine-7 or eFluor 450-conjugated antibodies (eBiosciences, San Diego, CA, USA) were used to stain cells. EFluor 450-CD3 (48–0038), PE-Cy7-CD4 (25–0049) and PE-pdpn (12–9381) were used for cell surface staining, according to the manufacturer’s instructions. PBMC were fixed and permeabilized for APC-IL-17A (17–7179) intracellular staining. Cells were incubated in cold PBS and cold 2 % paraformaldehyde in the dark during 1 h for fixation step. For permeabilization, cells were incubated in PBS with 0.2 % Tween at 37 °C for 15 min, before intracellular staining. Flow cytometry staining buffer from eBiosciences (San Diego, CA, USA) was used for staining protocol. Analysis was done with the Kaluza software (Beckman Coulter, Brea, CA, USA).

### Monocyte contribution

To remove monocytes, PBMC were pre-incubated during 2 h at 37 °C. The percentage of removal monocytes by adherence was verified with flow cytometry. This method allowed removing at least 50 % of monocytes.

### Inhibition of podoplanin

A dose–response curve was performed to determine the concentration of purified anti-human podoplanin antibody (14–9381, eBiosciences, San Diego, CA, USA) required for maximal inhibition. PBMC were pre-incubated for 4 h with different concentrations of anti-podoplanin (0, 1, 5, 10 and 20 μg/ml) before co-culture assay. According to the results of dose–response curve, all experiments studying podoplanin were realized with anti-podoplanin at 5 μg/ml. A control antibody against the BetV1 allergen with no effect in the assays was used at the same concentration (anti-BetV1 Ab, Dendritics, Lyon, France).

### Small interfering RNA (siRNA)

A mixture of four small interfering RNA (siRNA) provided as a single reagent (siGENOME human SMARTPool siRNA) specific for podoplanin (M-017560-01-0005) was purchased from Dharmacon, Lafayette, CO, USA. RA synoviocytes were seeded at a density of 5 × 10^5^ cells/12-well plate before transfection and used at 80–90 % confluence. Cells were transfected with control siRNAs (siCONTROL siRNA as a negative control (mock) and siGLO peptidylpropyl isomerase B (PPIB) (cyclophilin B) as a positive control) or target siRNAs (siGENOME SMARTPool pdpn siRNA) by nucleofection (Amaxa Pharma, London, UK) according to the manufacturer’s instructions (program U23; Human Dermal Fibroblast Nucleofector kit). Dose– and time–response experiments were performed to determine the best time point and the best suitable concentration of siRNA duplexes needed for efficacious RNA silencing. Cells were nucleofected with 37.6, 100 or 376 nM of pdpn siRNA (siPdpn), per 5 × 10^5^ cells. Twenty-four hours or 48 h post-transfection, RNA was extracted and RT-PCR performed to detect the RNA silencing. The best condition was at 100 nM, at 48 h, thus this condition was used for the presented experiments.

### RNA extraction and real-time PCR

Total RNA of synoviocytes was extracted using the RNeasy Mini Kit (Qiagen®, Hilden, Germany) and quantified with the Quant-it kit assay (Invitrogen™ by Thermo Fisher Scientific, Grand Island, NY, USA) following the manufacturer’s instructions. cDNA was synthesized using the QuantiTect reverse transcription kit (Qiagen®) according to the manufacturer’s instructions. SYBR green-based real-time qRT-PCRs were performed on the CFX96 Real-Time PCR Detection System (BioRad, Hercules, CA, USA) using the QuantiFast SYBR green kit and QuantiTect primers (Qiagen®). Cycle threshold values were normalized with respect to the endogenous control gene glyceraldehyde 3-phosphate dehydrogenase (GAPDH). The relative expression of pdpn and PPIB genes in the different conditions was determined using the comparative threshold cycle method as described by the manufacturer.

### Statistical analysis

Statistical analyses for co-culture assays were performed using two-way ANOVA. For transwell, monocytes and podoplanin experiments a paired nonparametric Wilcoxon test was used. All analyses were performed with GraphPad Prism 6 software (GraphPad Software, San Diego, CA, USA). *p* values less than or equal to 0.05 were considered as significant.

## Results

### Interaction between RA synoviocytes and PBMC induces IL-6 and IL-1β production

**Fig. 1:**
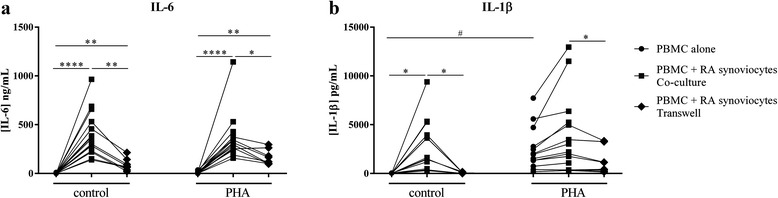
Effect of interaction between synoviocytes and PBMC on IL-6 and IL-1β production. Healthy PBMC were cultured alone or in co-culture with RA synoviocytes at a 5:1 ratio for 48 h, in the presence or absence of PHA (5 μg/ml). The transwell system was used in co-cultures to inhibit cell–cell contact. Production of IL-6 (**a**) and IL-1β (**b**) in cell supernatants was measured by ELISA. Each linked dot plot represents one experiment in the different conditions. ^*^Compares the culture conditions (PBMC alone, co-culture or transwell) with and without PHA. ^#^Compares the activation state (control or PHA) in the culture conditions. ^*#^ *p ≤* 0.05. *IL* interleukin, *PBMC* peripheral blood mononuclear cells, *PHA* phytohemagglutinin, *RA* rheumatoid arthritis

PBMC produce pro-inflammatory cytokines, such as IL-6 and IL-1β, which are implicated in the Th17 differentiation [16–18]. Resting PBMC alone produced IL-6 at low levels and their activation by PHA had a modest effect (1.4 ± 3.4 ng/ml vs. 13.4 ± 11.8 ng/ml, Fig. 1a). IL-1β production was almost undetectable in control condition (7.2 ± 16.1 pg/ml, Fig. 1b), and PHA activation highly increased its secretion (2630.1 ± 2397.3 pg/ml, *p* = 0.03, Fig. 1b). Co-culture of resting PBMC and synoviocytes significantly increased IL-6 and IL-1β production compared with PBMC alone (443.0 ± 240.7 ng/ml vs. 1.4 ± 3.4 ng/ml*; p* = 0.0001 and 2794.4 ± 2862.2 pg/ml vs. 7.2 ± 16.1 pg/ml; *p* = 0.02, respectively, Fig. 1). Activation of PBMC by PHA had no additional effect in co-culture (Fig. 1). These results indicated that the cell–cell contact was sufficient to activate the pro-inflammatory cytokine production. Furthermore, as PBMC and synoviocytes from different donors were used in the different experiments, this resulted in heterogeneity of cytokine production observed in Fig. 1.

To investigate the importance of cell–cell contact, a transwell system was used. The insert had a pore size of 0.4 μm, which prevents direct cell–cell contact but allows the exchange of soluble factors. In this transwell system, IL-6 and IL-1β production was significantly decreased compared to control (89.1 ± 58.6 ng/ml vs. 289.5 ± 130.9 ng/ml, *p* = 0.008 and 40.1 ± 46.3 pg/ml vs. 1488.9 ± 1505.2 pg/ml, *p* = 0.008, respectively, Fig. 1). A significant decrease of IL-6 and IL-1β production in the transwell system was also observed with T cell receptor (TCR) activation by PHA, nevertheless with a tendency to a lower effect than in the control condition. This confirmed that cell–cell contact was sufficient and required to activate cells to produce pro-inflammatory cytokines. Moreover, to reinforce this result, fixed synoviocytes were used in co-cultures. In these conditions, the secretion of IL-6 and IL-1β were also induced. Nevertheless, the production was at least 50 % lower for IL-6, as synoviocytes were fixed and the level of IL-1β produced mostly by monocytes was slightly decreased (data not shown).

### PBMC activation and cell interactions with synoviocytes are needed for a high IL-17 secretion

**Fig. 2:**
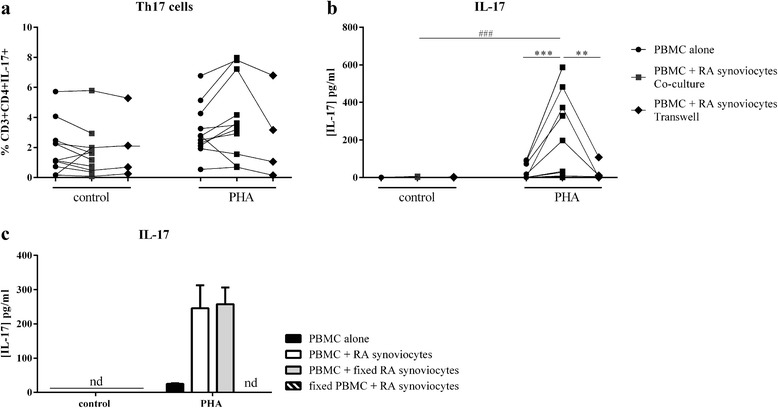
Effect of interaction between synoviocytes and PBMC on Th17 differentiation and IL-17 secretion. Healthy PBMC were cultured alone or in co-culture with RA synoviocytes at a 5:1 ratio for 48 h, in the presence or absence of PHA (5 μg/ml). The transwell system was used in co-cultures to inhibit cell–cell contact and fixed synoviocytes or fixed PBMC were used. PBMC were recovered after 48 h and stained for the surface markers CD3 and CD4, and intracellular IL-17A. The percentage of CD3 + CD4 + IL-17A + is represented for each experiment (**a**). IL-17A production was measured in supernatants after 48 h, by ELISA (**b** and **c**). Each linked dot plot represents one experiment in the different conditions. ^*^Compares the culture conditions (PBMC alone, co-culture or transwell) with and without PHA. ^#^Compares the activation state (control or PHA) in the culture conditions. ^*#^ *p ≤* 0.05. *IL* interleukin, *PBMC* peripheral blood mononuclear cells, *PHA* phytohemagglutinin, *RA* rheumatoid arthritis

Th17 cells have been identified as a major source of IL-17 [19] and IL-6 and IL-1β, which are increased by cell interactions (Fig. 1) that are critical for Th17 differentiation [16–18]. The role of cell interactions on the IL-17 pathway was studied to distinguish the intracellular staining from the secretion of IL-17 in medium. Flow cytometry analysis showed that IL-17-positive cells were observed in culture of PBMC alone or in co-culture with synoviocytes. However, there was no difference in the percentage of IL-17-positive T cells in resting PBMC alone or cultured with synoviocytes (1.9 ± 1.7 % vs. 1.7 ± 1.6 %, respectively, Fig. 2a). The effect of PHA activation was not significant but with a tendency to higher Th17 cells with PHA (PBMC alone: 1.9 ± 1.7 % vs. 3.1 ± 1.7 %; co-culture: 1.7 ± 1.6 % vs. 3.9 ± 2.7 %, *p* = 0.06, Fig. 2a). In addition, PHA activation also increased the percentage of IL-17+ cells among the CD3 + CD4- in PBMC alone (1.1 ± 0.6 % vs. 1.8 ± 0.9 %, *p* = 0.053) and in co-culture (1.1 ± 0.7 % vs. 2.1 ± 0.7 %, *p* = 0.01); but there was no difference in the percentage between PBMC alone and co-culture (data not shown).

Actual IL-17 secretion in supernatants was measured by ELISA. Without PHA, IL-17 production was undetectable in resting PBMC (Fig. 2b); but it was present at a very low level in co-culture of PBMC and synoviocytes (1.1 ± 2.2 pg/ml). TCR activation by PHA did not increase significantly IL-17 secretion in PBMC alone (Fig. 2b). In contrast, there was a significant increased production of IL-17 in co-culture with activated PBMC (1.1 ± 2.2 pg/ml vs. 185.5 ± 220.3 pg/ml, *p* = 0.002, Fig. 2b). The activation of PBMC by anti-CD3 and anti-CD28 gave similar results as PHA activation (data not shown). These results demonstrated that the combination of TCR activation and cell–cell contact was required to obtain a high IL-17 secretion. Furthermore, TNF, which is a major cytokine involved in RA pathogenesis, is known to stimulate synoviocytes. Activation of synoviocytes by TNF was tested in co-culture without TCR stimulation, giving similar results than in the control condition (data not shown).

To confirm the crucial role of cell–cell contact in IL-17 production, the transwell system was used. This contact inhibition had no effect on Th17 differentiation, as the percentage of IL-17 positive cells was similar between co-culture and transwell system (2.1 ± 2.6 % vs. 2.1 ± 2.3 % without PHA; 4.3 ± 3.7 % vs. 2.8 ± 3.0 % with PHA, Fig. 2a). Conversely, without cell interactions in the PHA activation condition, IL-17 secretion was strongly reduced (265.0 ± 183.9 pg/ml vs. 30.5 ± 44.6 pg/ml, *p* = 0.008), reaching the same level as PBMC alone (30.5 ± 51.5 pg/ml vs. 26.3 ± 36.7 pg/ml, respectively, Fig. 2b). This transwell experiment clearly demonstrated that direct cell interactions between activated PBMC and RA synoviocytes were crucial for high levels of IL-17 secretion. Furthermore, using fixed synoviocytes with live activated PBMC induced IL-17 secretion at a similar level compared to co-culture with nonfixed synoviocytes. Conversely, fixed PBMC with live synoviocytes produced no IL-17 (Fig. 2c).

### Co-culture between autologous cells also increases pro-inflammatory cytokine production

**Fig. 3:**
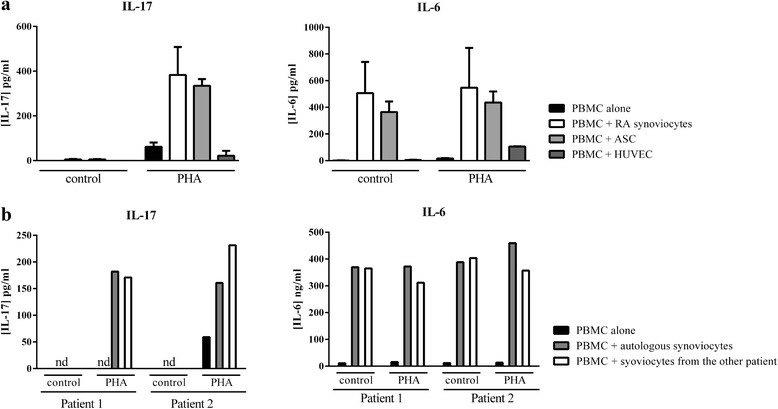
Confirmation in an autologous system and with other mesenchymal cell types. Healthy PBMC were cultured alone or in co-culture with RA synoviocytes, ASC or HUVEC at a 5:1 ratio for 48 h, in the presence or absence of PHA (5 μg/ml). Levels of IL-17 and IL-6 were measured in the supernatants after 48 h by ELISA (**a**). PBMC from RA patients were cultured alone or in co-culture with autologous synoviocytes or from other patients, at the ratio 5:1 for 48 h, in the presence or absence of PHA (5 μg/ml). Levels of IL-17 and IL-6 were measured in the supernatants after 48 h by ELISA. Values are individual results (**b**). *ASC* adipose-derived stem cells, *HUVEC* human umbilical vein endothelial cells. *IL* interleukin, *PBMC* peripheral blood mononuclear cells, *PHA* phytohemagglutinin, *RA* rheumatoid arthritis

The cell specificity of IL-17 production induced by cell interactions between immune and mesenchymal cells was studied by comparing different mesenchymal cell types (synoviocytes and ASC) and endothelial cells (HUVEC) in co-culture. As shown in Fig. 3a, with both RA synoviocytes and ASC, interactions with PBMC induced high IL-6 production and a high IL-17 secretion in co-culture with activated PBMC (Fig. 3a). In contrast, co-culture with HUVEC did not induce IL-6 and IL-17 production (Fig. 3a). This indicated that specific interactions between fibroblast-like cells and immune cells are critical to induce high pro-inflammatory cytokine production.

To confirm that pro-inflammatory cytokine production resulting from cell interactions may occur inside the inflamed synovium, co-culture experiments with synoviocytes and PBMC from the same RA patient were tested. As observed in Fig. 3b, co-cultures with autologous cells gave similar results as co-cultures with RA synoviocytes and healthy PBMC. This indicated the absence of contribution of alloreactivity in the effect. Indeed, cell interactions were sufficient to induce IL-6 (Fig. 3b). IL-17 was markedly more produced in co-culture with autologous activated PBMC (Fig. 3b). In parallel, co-cultures between PBMC from patient 1 and synoviocytes from patient 2 and the other way around were tested. Results were similar in both systems (Fig. 3b) indicating the critical role of cell interactions in the pro-inflammatory cytokine production.

### Monocytes do not contribute to the high IL-17 production

**Fig. 4:**
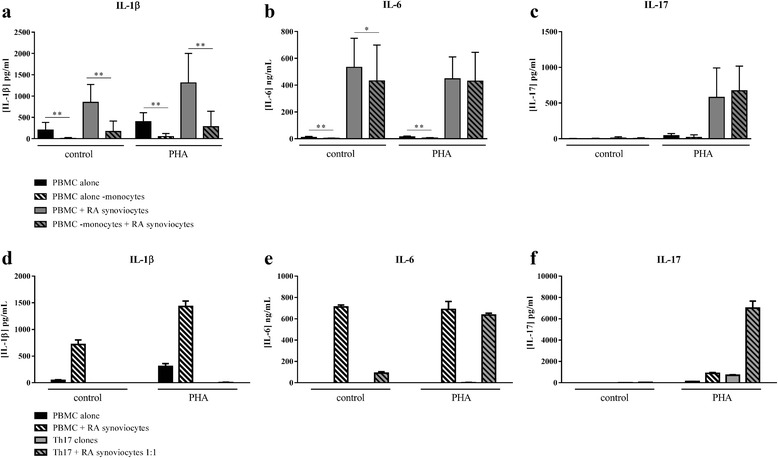
Role of monocytes on pro-inflammatory cytokine production. Healthy PBMC or Th17 clones were cultured alone or in co-culture with RA synoviocytes at a 5:1 ratio or 1:1 ratio, respectively, for 48 h, in the presence or absence of PHA (5 μg/ml), and monocytes were removed or not by adherence before culture. Production of IL-1β (**a**, **d**), IL-6 (**b**, **e**) and IL-17 (**c**, **f**) were measured in supernatants after 48 h, by ELISA. ^*^Compared conditions with and without monocytes. ^*^ *p ≤* 0.05. *IL* interleukin, *PBMC* peripheral blood mononuclear cells, *PHA* phytohemagglutinin, *RA* rheumatoid arthritis

Considering the role of IL-6 and IL-1β in the Th17 pathway and the role of cell interactions in maintaining inflammation, the potential contribution of monocytes in this loop was investigated. To study their involvement in our co-culture system, monocytes were removed by adherence. As IL-1β is mainly produced by monocytes in PBMC, the reduction of IL-1β production can be considered as a good marker for the removal of monocytes. As observed in Fig. 4a, the production of IL-1β was indeed significantly inhibited in all conditions without monocytes (*p* = 0.004). In contrast, IL-6 is a pro-inflammatory cytokine produced by many cell types, including PBMC and synoviocytes. In control condition, IL-6 secretion was significantly decreased, but less than IL-1β, in culture of PBMC alone and in co-culture without monocytes (10.3 ± 8.0 ng/ml vs. 3.4 ± 2.5 pg/ml, *p* = 0.003; 532.1 ± 217.5 ng/ml vs. 431.7 ± 267.4 ng/ml, *p* = 0.01, respectively, Fig. 4b). With PHA, removal of monocytes had an effect only in PBMC alone (14.5 ± 6.1 ng/ml vs. 6.5 ± 2.3 ng/ml, *p* = 0.003, Fig. 4b). Surprisingly, IL-17 production was not affected by removing monocytes (Fig. 4c). These results showed that monocytes have no major role in the high IL-17 secretion during cell interactions, indicating the involvement of key interactions between lymphocytes and mesenchymal cells.

To confirm the crucial role of synoviocytes and Th17 cells in the high IL-17 secretion, co-cultures between synoviocytes and Th17 clones (ratio 1:1) were performed. As observed in Fig. 4d, there was no IL-1β production compared to co-cultures with PBMC. This result was expected as the major source of IL-1β was not present. In co-cultures with Th17 clones, IL-6 secretion was induced in control condition as with PBMC, even the level of production was lower than with PBMC (90.2 ± 10.0 pg/ml vs. 712.1 ± 12.5 pg/ml), and with Th17 clones, PHA activation increased IL-6 secretion (635.6 ± 12.5 pg/ml vs. 90.2 ± 10.0 pg/ml, Fig. 4e). As with PBMC, the detection of IL-17 production was possible only with PHA activation (701.7 ± 39.1 pg/ml vs. 15.2 ± 0.2 pg/ml, Fig. 4f) and the interaction with synoviocytes largely increased this secretion (7013.0 ± 458.5 pg/ml vs. 701.7 ± 39.1 pg/ml, Fig. 4f). These results confirmed the crucial role of TCR activation and of cell–cell contact in the high IL-17 production and make synoviocytes and Th17 cells the two major cell types involved in this elevated secretion.

### Podoplanin plays a major role in high IL-17 secretion during co-culture between activated PBMC and RA synoviocytes

**Fig. 5:**
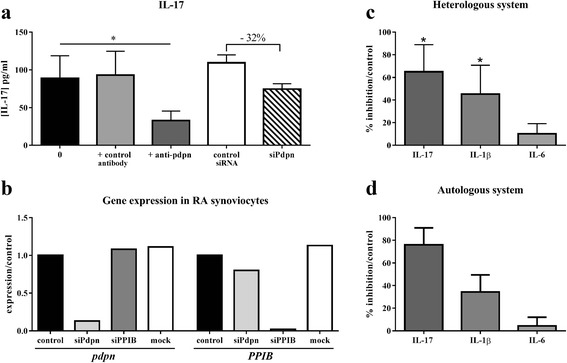
The major role of podoplanin in the high IL-17 secretion. Healthy or RA PBMC were pre-incubated 4 h with or without human anti-pdpn or irrelevant antibody before co-culture with synoviocytes or autologous synoviocytes, respectively, for 48 h, in the presence of PHA (5 μg/ml). For siRNA, RA synoviocytes were transfected without (control) or with siRNA (pdpn, PPIB or mock) and then used in co-culture with healthy PBMC, in the presence of PHA, during 48 h. Levels of IL-17, IL-1β and IL-6 were measured in the supernatants after 48 h by ELISA. The concentration of IL-17 in the different conditions of culture is represented (**a**). The expression of the genes *pdpn* and *PPIB* compared to control (control = 1) (**b**) is represented as well as the percentage of cytokine inhibition compared to control in the heterologous system (**c**) and in the autologous system (**d**). * *p ≤* 0.05 compared to control. *IL* interleukin, *PBMC* peripheral blood mononuclear cells, *PHA* phytohemagglutinin, *RA* rheumatoid arthritis, *pdpn* podoplanin, *PPIB* peptidylpropyl isomerase B, *siRNA* small interfering RNA

The role of direct physical cell interactions in the high IL-17 production is critical. As podoplanin (pdpn) can be expressed by different cell types, including synoviocytes, its potential role was studied with a blocking anti-pdpn antibody. A dose–response curve was performed with different concentrations of anti-pdpn antibody (Ab), 0, 1, 5, 10 and 20 μg/ml, to determine the optimum concentration of anti-pdpn Ab. The concentration of 5 μg/ml of antibody pre-incubated for 4 h gave the higher inhibition of cytokine production (data not shown). In the co-culture of synoviocytes and activated PBMC, the presence of anti-pdpn Ab inhibited significantly IL-17 secretion by 64.9 ± 24.0 %. (*p* = 0.008, Fig. 5a and c), IL-1β secretion (45.3 ± 25.5 % of inhibition, *p* = 0.02, Fig. 5c), but not IL-6 secretion (10.1 ± 9.0 % of inhibition, *p* = 0.25, Fig. 5c). Moreover, the effect of anti-pdpn antibody was specific as results with a control antibody were similar than without antibody (93.2 ± 31.4 pg/ml vs. 88.8 ± 29.9 pg/ml for IL-17, Fig. 5a, data not shown for IL-1β and IL-6).

The inhibition of pdpn was tested by siRNA specific for pdpn in synoviocytes. The presence of siPdpn inhibited the IL-17 production by around 30 % (74.6 ± 7.0 vs. 109.3 ± 10.5 pg/ml, Fig. 5a). This effect was specific for siPdpn as there was no inhibition of IL-17 secretion with the mock and with siPPIB (103.2 ± 5.9 pg/ml and 112.5 ± 15.6 pg/ml vs. 109.3 ± 10.5 pg/ml, respectively, data not shown). Furthermore, to verify the specificity of the siRNA, the gene expression of pdpn and PPIB was tested. In Fig. 5b, the expression of pdpn was inhibited only with the siPdpn, but not with the siPPIB (positive control) neither with the mock (negative control). The expression of PPIB was inhibited with the siPPIB but not with the siPdpn neither with the mock. This confirmed the specificity of the siPdpn.

Furthermore, with a therapeutic perspective in mind, anti-pdpn Ab was tested in an autologous system. As observed in Fig. 5d, the presence of anti-pdpn Ab decreased IL-17 (76.0 ± 26.0 % of inhibition) and IL-1β production (34.3 ± 26.2 % of inhibition), in a similar percentage as in the heterologous system. IL-6 secretion was not affected (Fig. 5d). These results in the autologous system supported the involvement of pdpn in the high IL-17 secretion during cell interactions as seen in vivo.

**Fig. 6:**
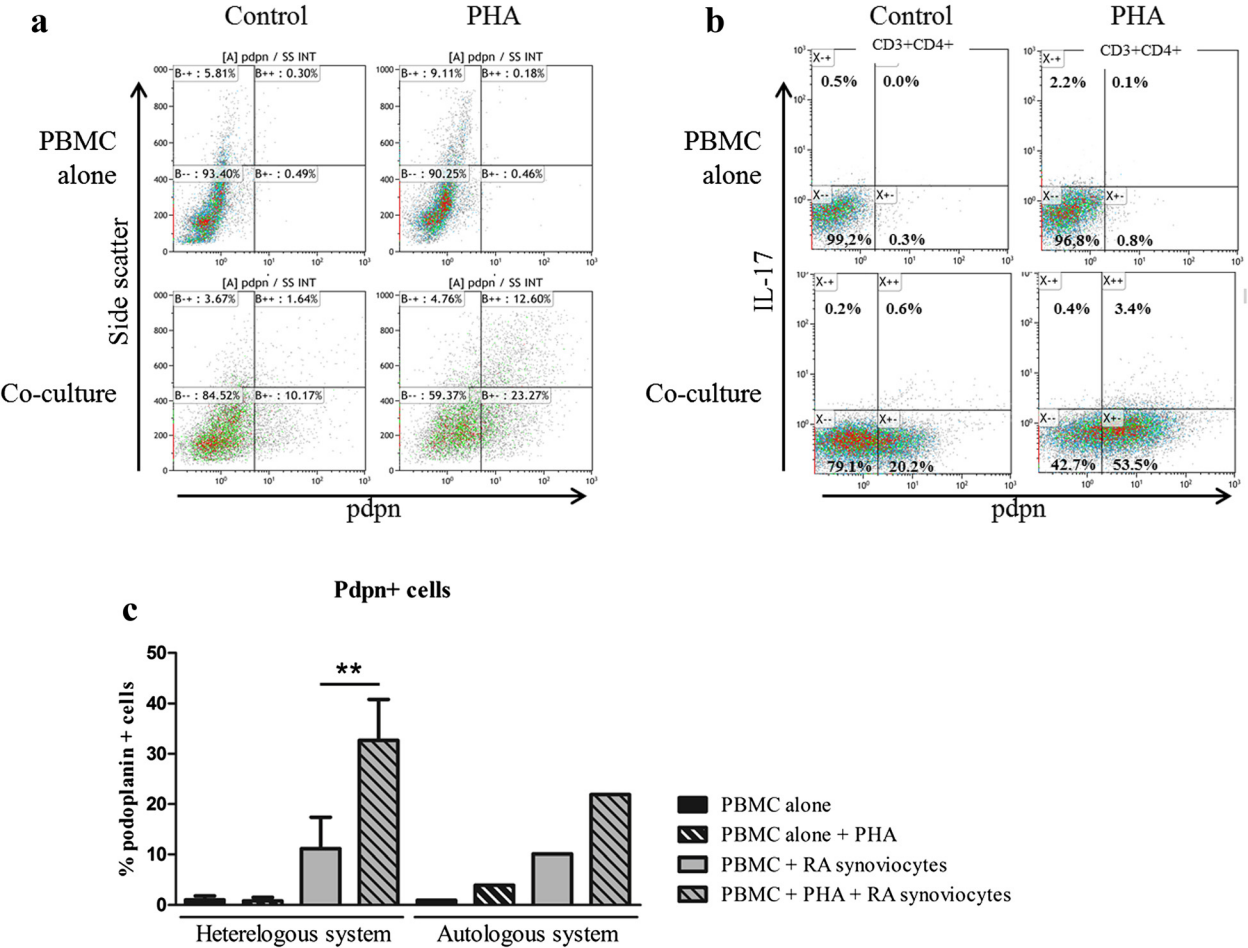
Expression of podoplanin in PBMC alone or in co-culture. Healthy PBMC were cultured alone or in co-culture with RA synoviocytes at a 5:1 ratio for 48 h, in the presence or absence of PHA (5 μg/ml). Cells were recovered after 48 h and stained for pdpn (**a** and **c**) or CD3, CD4, IL-17 and pdpn (**b**). Dot plot of one experiment is represented (**a** and **b**). The percentage of pdpn + cells in the different conditions is represented (**c**). ^*^ *p ≤* 0.05. *IL* interleukin, *PBMC* peripheral blood mononuclear cells, *PHA* phytohemagglutinin, *pdpn* podoplanin, *RA* rheumatoid arthritis

Pdpn has been shown to be expressed mainly by RA synoviocytes. To study the regulation of its expression during cell interactions, co-cultures were performed as described before and after 48 h, cells (synoviocytes and PBMC) were recovered and stained for pdpn. PBMC alone showed a very low percentage of pdpn + cells (1.0 ± 0.8 % without PHA; 0.8 ± 0.7 % with PHA, Fig. 6a and c). As synoviocytes expressed pdpn, the side scatter was focus more for PBMC. Interestingly, the percentage of pdpn + cells increased in co-culture (11.2 ± 6.2 % without PHA; 32.7 ± 8.1 % with PHA, *p* = 0.04, Fig. 6a and c). This increase was present in different populations, CD3- (9.2 ± 2.2 % without PHA; 35.9 ± 0.5 % with PHA); CD3 + CD4- (11.3 ± 3.7 % without PHA; 43.7 ± 7.2 % with PHA) and in CD3 + CD4+ (17.1 ± 5.1 % without PHA; 55.7 ± 1.5 % with PHA). Interestingly, in CD3 + CD4+, co-culture increased the pdpn expression in IL-17- (0.9 ± 0.6 % vs. 16.7 ± 5.0 % without PHA; 3.3 ± 3.4 % vs. 54.3 ± 0.9 with PHA, Fig. 6b) but this effect was major in IL-17+ cells (9.4 ± 1.1 % vs. 69.7 ± 7.6 % without PHA; 8.4 ± 8.3 % vs. 85.7 ± 2.8 % with PHA). These results indicated that pdpn expression could be regulated by cell–cell contact, with an effect mainly in Th17 cells. These results were also consistent with the increase of IL-17 secretion associated with overexpression of pdpn in both synoviocytes and activated PBMC. In addition, PBMC showed an increased size in co-culture, especially with PHA (data not shown), reflecting the high size with the plasma cell morphology observed previously in IL-17+ cells under activation and in in vivo [3].

## Discussion

Cell interactions between mesenchymal and immune cells are known to induce the production of pro-inflammatory cytokines and also to affect the survival of both cell types [7, 20–22]. In the context of RA, a co-culture system between RA synoviocytes and PBMC was used to mimic the in vivo situation. Cell interactions were sufficient to provide a necessary activation state for the secretion of IL-6 and IL-1β and this is in agreement with a previous study showing that MSC-PBMC interactions increased IL-6 and IL-1β mRNA [7]. These observations reflect how high levels of pro-inflammatory cytokines, including IL-6 and IL-1β, can be produced locally by the RA synovium [23]. Furthermore, co-culture with autologous cells, PBMC and synoviocytes from the same patient, validated our co-culture in vitro model mimicking the in vivo inflammation.

IL-6 and IL-1β are known to be involved in Th17 cell differentiation [16–18]. Th17 cells in turn secrete IL-17 which acts on synoviocytes, often in synergy with other cytokines such as TNF-α, IL-1β or granulocyte-macrophage colony-stimulating factor (GM-CSF) [2, 24–26]. Considering the results on IL-6 and IL-1β production, the effect of cell interactions on the Th17 pathway was studied to differentiate the intracellular expression from the secretion of IL-17. The percentage of CD3 + CD4 + IL-17+ cells was evaluated in PBMC alone or in co-culture. Cell contact alone had no major effect on Th17 differentiation measured by intracellular staining. Only TCR activation had a modest effect. This indicated that Th17 differentiation requires cell activation more than cell–cell contact.

When looking at the production of IL-17 and in contrast to that of IL-6 and IL-1β, cell interactions were not sufficient to induce a high IL-17 secretion. Its production required two signals, TCR activation and cell–cell contact. Moreover, activation of synoviocytes by TNF alone in co-culture without TCR stimulation had no effect on IL-17 production. In fact, IL-17 secretion during cell interactions was dependent on T cell but not synoviocyte activation. Transwell experiments confirmed that cell interactions were crucial to have an elevated IL-17 secretion even in the presence of TCR activation.

These results reveal a major discrepancy between intracellular and secreted IL-17. The intracellular presence of IL-17 inside Th17 cells does not mean by itself a high secretion of IL-17. The presence of Th17 cells even with TCR activation was not enough to have the release of high levels of IL-17. It was necessary that activated cells could physically interact with mesenchymal cells, derived from either synovium, bone marrow, or adipose tissue. Thus, TCR activation and cell–cell contact are two needed mechanisms leading to a high IL-17 production and this differs from intracellular expression of IL-17. Both mechanisms are present during RA pathogenesis [13, 27–30], and this could explain the presence of IL-17 in the joints of RA patients [31, 32].

Furthermore, the secretion of IL-17 was variable depending on experiments which used different donors for PBMC and RA synoviocytes. This variability reflected the heterogeneity which characterizes the RA population suggesting the variable contribution of IL-17 in the inflammatory process. The variability in IL-17 secretion observed in our experiments could explain in part the heterogeneity of the response to an anti-IL-17 treatment in RA patients [33, 34]. Such heterogeneity remains to be explained. One explanation could be the contribution of gene polymorphisms in the regulation of the Th17 pathway. *IL4R* gene polymorphisms have been associated with RA severity by increasing the Th17 cell frequency and by modulating the inhibitory effect of IL-4 on Th17 development [35] and the modulation of IL-17 production [36]. *IL-23R* polymorphisms have been implicated in IL-17A expression in RA [37].

The critical contribution of interactions between immune cells and mesenchymal cells indicated the need to identify a molecular mechanism. The limited contribution of monocytes suggested a molecule present on lymphocytes or on mesenchymal cells or on both. Pdpn, which is a type I transmembrane protein, appeared as a good candidate. Pdpn-mediated interaction of RA synoviocytes and platelets modulates IL-8 production [8]. Furthermore, in the SKG spontaneously occurring arthritis model, pdpn is upregulated in Th17 cells compared to other Th cell subsets [9] and in the synovium of RA patients [11]. In a mouse model of multiple sclerosis, mice treated with anti-pdpn present a delayed onset of symptoms [10]. Based on these observations, an antibody against pdpn was used in the co-culture system and siRNA specific for pdpn was used on synoviocytes. Both means induced an inhibition of IL-17 production and confirmed the role of pdpn in the IL-17 secretion.

These results focused on the podoplanin expressed by RA synoviocytes but it was known that Th17 cells could express pdpn, notably in an experimental arthritis model and in clinical RA [38, 39]. In accordance with this, the three different tested protocols, the pre-incubation of PBMC, the pre-incubation of synoviocytes or the pre-incubation of both cells, gave similar results (data not shown). Acting first on synoviocytes or PBMC did not affect the inhibitory effect of the anti-pdpn. This is in line with the expression of pdpn by Th17 cells and the fact that the effect of anti-pdpn could be both direct on Th17 cells and indirect by acting on synoviocytes to inhibit the IL-17 production during cell interactions. In addition, the lower percentage of inhibition obtained with siPdpn compared with the anti-pdpn Ab could also indicate the respective involvement of pdpn expressed by synoviocytes and by Th17 cells. The regulation of pdpn in PBMC and specifically in Th17 cells remains to be clarified.

The interaction between pdpn and its receptor could occur in the two directions, from synoviocytes to PBMC, or from PBMC to synoviocytes. The receptor of pdpn CLEC-2 is known to be mainly expressed by platelets [40] and also by mature dendritic cells or peripheral blood B lymphocytes [41–44]. Its expression in our co-culture system could be studied to provide a new insight on the pathway by which pdpn could influence the IL-17 secretion. Currently, there is no evidence for its expression on Th17 cells and this could also suggest a possible new receptor for pdpn. A recent study has shown that pdpn can negatively regulate CD4+ effector T cell functions through pdpn-CLEC-2 interaction [45]. A high pdpn expression was found on “nonpathogenic” Th17 lymphocytes while “pathogenic” Th17 cells expressed less pdpn. Thus, pdpn displays two divergent functions which may depend on different ligands. One ligand, such as CLEC-2 could mediate T cell inhibition while another ligand could promote inflammation by stimulating pro-inflammatory cytokine production. Furthermore, our results demonstrated that the inhibition of the interaction mediated by pdpn decreased at least by 50 % the secretion of IL-17 and of IL-1β, but not that of IL-6. Furthermore, in both PBMC and synoviocytes, pdpn expression was increased in co-culture with TCR activation which correlates with the high IL-17 production. These results suggested that cell interactions of synoviocytes with activated immune cells increased pdpn expression that contributed to the high IL-17 secretion.

If podoplanin seems to be a good potential therapeutic target, investigating the effect of cell interactions on other signaling molecules involved in Th17 differentiation and function could be interesting. Indeed, the interaction with mesenchymal stem cells (MSC) could on the one hand enhance Th17 activity [7, 46] but on the other hand it could repress Th17 molecular program through PD-1 [47]. Furthermore, IL-17A can induce soluble PD-1 (sPD-1) which level is increased in RA serum. This overexpression of sPD-1 might block the inhibitory PD-1 pathway [48]. Investigating the PD-1 pathway in co-culture system could allow identifying another mechanism. Signaling lymphocytic activation molecule (SLAM) is another candidate as it promotes the differentiation of IL-17-secreting effectors [49]. Inducible T cell costimulator (ICOS) signaling, which belongs to the CD28 costimulatory molecule superfamily, has been also shown to play a critical role in the generation and maintenance of human Th17 cells [50] and it could be another candidate.

## Conclusions

This study showed that cell interactions between fibroblast-like mesenchymal cells and immune cells play a major role in pro-inflammatory cytokine production, leading to a major increase in IL-17 secretion distinct from intracellular expression. The interaction molecule podoplanin appears to have a large contribution to the high IL-17 secretion that in turn may contribute to the chronicity of inflammation. In this context, pdpn could be a potential therapeutic target to block Th17 cell activity during chronic inflammation.

## Abbreviations

ASC, adipose-derived stem cells; ELISA, enzyme-linked immunosorbent assay; GAPDH, glyceraldehyde-3-phosphate dehydrogenase; HUVEC, human umbilical vein endothelial cells; IFN, interferon; IL, interleukin; MSC, mesenchymal stem cells; PBMC, peripheral blood mononuclear cells; PHA, phytohemagglutinin; pdpn, podoplanin; PPIB, peptidylpropyl isomerase B; RA, rheumatoid arthritis; siRNA, small interfering RNA; TCR, T cell receptor; TNF, tumor necrosis factor
